# Editorial: Social determinants and psychosocial factors that impact on health status

**DOI:** 10.3389/fpsyg.2024.1405206

**Published:** 2024-04-26

**Authors:** Rubén González-Rodríguez, Manuel Gandoy-Crego, Teresa Vilaça

**Affiliations:** ^1^Universidade de Vigo, Grupo de Estudos en Traballo Social: Investigación e Transferencia (GETS-IT), Ourense, Spain; ^2^Social Work Studies Group: Research and Transfer (GETS-IT), Galicia Sur Health Research Institute (IIS Galicia Sur), SERGAS-UVIGO, Vigo, Spain; ^3^Department of Psychiatry, Radiology, Public Health, Nursing and Medicine, University of Santiago de Compostela, Santiago de Compostela, Spain; ^4^Institute of Education at the University of Minho, Universidade do Minho, Braga, Portugal

**Keywords:** social determinants, psychosocial factors, health, wellbeing, behavior, health education

Historically, several conceptual frameworks have been proposed to explain the determinants affecting the health-disease continuum. All of them considered individual variables, lifestyle, and health systems, but also contextual variables. Currently, there is consensus on the importance of the impact of social conditions, such as working conditions, socio-environmental setting, income level, access to education, or political-economic variables that play a crucial role in determining the health status of the population. To address these concerns, the World Health Organization (WHO) defines the social determinants of health as the “structural factors and living conditions that are responsible for much of the health inequities (…). The term ≪social determinants≫ summarizes the set of social, political, economic, environmental, and cultural factors that have a strong influence on health status” (WHO, 2008).

All countries have social inequalities in health, and they occur gradually along the social scale. The impact of these disparities is significant, and their trend is increasing. However, it seems that the greater the social disadvantage in any of the social determinants, the worse the health outcomes, and the worse when several axes of inequality concur (Ruiz et al., 2022). However, there is enough evidence to demonstrate how the implementation of appropriate health and social policies can reduce these health disparities (Benach, 1997), establishing strategies that consider these social inequalities in a multidisciplinary manner and focusing actions on primary interventions and health promotion (De La Guardia and Ruvalcaba, 2020).

This set of social factors and exposure to them condition the health status of the individual and his or her social participation in the community. From variables linked to the labor context, such as unemployment, which is associated with greater cardiovascular risk factors, especially in young people (Vancea and Utzet, 2017), or mental illness (Frasquilho et al., 2015). The physical environment in which we live also influences our state of health and enhances (or diminishes) the healthy lifestyle habits that individuals develop (Twohig-Bennett and Jones, 2018). Also, the public policies implemented have led to a decrease in citizen participation, as well as to the exclusion of many people, depriving them of the right to health (Falkenbach and Greer, 2018). Other variables linked to the generation of social inequalities in health deserve special attention, such as gender or ethnicity. When we refer to gender, we are not referring to physical differences related to sex, but to the social inequalities in health that gender entails, such as inequalities to enjoy good health. These inequalities seem to persist even in crises, such as the COVID-19 pandemic (Zwar et al., 2023). This Research Topic aims to shed light on how psychosocial and contextual factors determine people's wellbeing and quality of life.

From a cultural perspective, Li et al. found gender differences in binge eating behavior. Their results indicated that, in Chinese culture, body dissatisfaction and self-acceptance, independently or through a serial form, mediate gender differences in binge eating behavior. Shin and Park also addressed gender, in this case, linked to the existence and quality of social networks, examining their associations with various impacts on physical and mental health and analyzing the results according to gender. The findings suggest that women benefit more from support networks and are also more vulnerable to network deficits.

The family and residential context was also a focus of interest. A narrative review (Faraji et al.) updates the available evidence on how different family-related factors are related to the fear of cancer recurrence among survivors. This research made it possible to categorize them into four factors: partner-related, parenting-related, family-related, and social interactions. Their categorization makes it possible to construct a more comprehensive model that helps healthcare personnel improve the design of family interventions. The family and communication with their cancer patient relatives was the subject of interest for Naghavi et al.. They analyzed the general and individual attitudes of caregivers and non-caregivers regarding communication with cancer patients. Their results noted the contrast between positive attitudes toward direct communication and the actual practices observed. They suggest the creation of protocols for conveying bad news in a culturally competent manner and facilitating the patient's need to express their emotions and needs. Melero et al. were concerned with the psychological wellbeing of adults raised in foster families. They found that there is no direct relationship between aging and a decrease in psychological wellbeing. Increasing age is only related to lower psychological wellbeing in the case of a lack of mastery of certain developmental tasks of adulthood.

Several studies addressed psychosocial variables closely linked to contextual elements of the health systems. Bayraktar and Ozkan studied posttraumatic growth, coping, and illness perceptions in cancer patients. They highlight the need to strengthen positive coping methods and implement interventions targeting the cognitive aspects of their illness perceptions. Their results indicate that the relevant variables affecting posttraumatic growth in cancer patients in different cultures do not change. Jeon and Noh analyze psychosocial factors associated with health behaviors in older pregnant women to identify which behaviors promote and harm health in the Korean context. Among the psychosocial factors that explained prenatal health-promoting behaviors were maternal-fetal attachment and the social atmosphere of pregnancy stress. In contrast, artificial conception, multipara, and maternal role stress influenced prenatal health-damaging behaviors.

The instruments used in the health system have been another focus of interest. Norouzkhani et al. were concerned about the information and support provided to patients with inflammatory bowel disease. Through a Delphi consensus study, they generated a questionnaire of 100 items grouped into three categories: support needs, sources of information, and specific information needs. Buki et al. warn of the difficulties in assessing psychosocial factors associated with colorectal cancer. As a solution, they develop and validate the psychometric properties of the Colorectal Cancer Literacy Scale-Uruguay Version, that assess culturally based factors that influence colorectal cancer screening behaviors.

Abudoush et al. analyze the lived experiences of chronic pain in Arabic-speaking populations and its relationship with psychosocial processes such as care, coping, or social support. It addresses realities in sociocultural settings other than the Western setting and provides conclusions that justify the development of culturally sensitive interventions. Zhou et al. also considered ethnic differences, analyzing the role of perceived discrimination as a mediator between cultural identity and mental health symptoms in adults. They highlight the need to consider racial, ethnic, and socioeconomic inequalities, as well as cultural identity and bias, in mental health research and interventions.

Concern for the more structural social determinants, specifically those affecting the health and wellbeing of the elderly, was addressed by Zhang et al.. They refer to the following primary structural indicators: socioeconomic development factors, political factors, environmental factors, and cultural factors. Their findings show that individual social factors alone are insufficient to achieve high levels of health in older adults.

This Research Topic covers a wide range of social determinants and psychosocial factors that affect health and wellbeing, including socioeconomic context, culture, family, and gender, among others. The scientific contributions of the subject suggest that approaches to health interventions should consider these social variables that have an impact on health. The findings provide important information for families, patients, healthcare professionals, and policymakers.

## Author contributions

RG-R: Conceptualization, Supervision, Writing – original draft, Writing – review & editing. MG-C: Conceptualization, Writing – review & editing. TV: Conceptualization, Writing – review & editing.
